# Adrenomedullin Expression Characterizes Leukemia Stem Cells and Associates With an Inflammatory Signature in Acute Myeloid Leukemia

**DOI:** 10.3389/fonc.2021.684396

**Published:** 2021-06-02

**Authors:** Giorgia Simonetti, Davide Angeli, Elisabetta Petracci, Eugenio Fonzi, Susanna Vedovato, Alessandra Sperotto, Antonella Padella, Martina Ghetti, Anna Ferrari, Valentina Robustelli, Rosa Di Liddo, Maria Teresa Conconi, Cristina Papayannidis, Claudio Cerchione, Michela Rondoni, Annalisa Astolfi, Emanuela Ottaviani, Giovanni Martinelli, Michele Gottardi

**Affiliations:** ^1^ Biosciences Laboratory, IRCCS Istituto Romagnolo per lo Studio dei Tumori (IRST) “Dino Amadori”, Meldola, Italy; ^2^ Unit of Biostatistics and Clinical Trials, IRCCS Istituto Romagnolo per lo Studio dei Tumori (IRST) “Dino Amadori”, Meldola, Italy; ^3^ Department of Clinical and Experimental Medicine, University of Padova, Padua, Italy; ^4^ Hematology and Transplant Center Unit, Dipartimento di Area Medica (DAME), Udine University Hospital, Udine, Italy; ^5^ IRCCS Azienda Ospedaliero-Universitaria di Bologna, Istituto di Ematologia “Seràgnoli”, Bologna, Italy; ^6^ Dipartimento di Medicina Specialistica, Diagnostica e Sperimentale, Università di Bologna, Bologna, Italy; ^7^ Department of Pharmaceutical and Pharmacological Sciences, University of Padova, Padua, Italy; ^8^ Hematology Unit, IRCCS Istituto Romagnolo per lo Studio dei Tumori (IRST) “Dino Amadori”, Meldola, Italy; ^9^ Hematology Unit & Romagna Transplant Network, Ravenna Hospital, Ravenna, Italy; ^10^ “Giorgio Prodi” Cancer Research Center, University of Bologna, Bologna, Italy; ^11^ Department of Morphology, Surgery and Experimental Medicine, University of Ferrara, Ferrara, Italy; ^12^ Scientific Directorate, IRCCS Istituto Romagnolo per lo Studio dei Tumori (IRST) “Dino Amadori”, Meldola, Italy; ^13^ Onco Hematology, Department of Oncology, Veneto Institute of Oncology IOV, IRCCS, Padua, Italy

**Keywords:** acute myeloid leukemia, adrenomedullin, hematopoiesis, inflammation, leukemia stem cells

## Abstract

Adrenomedullin (ADM) is a hypotensive and vasodilator peptide belonging to the calcitonin gene-related peptide family. It is secreted *in vitro* by endothelial cells and vascular smooth muscle cells, and is significantly upregulated by a number of stimuli. Moreover, ADM participates in the regulation of hematopoietic compartment, solid tumors and leukemias, such as acute myeloid leukemia (AML). To better characterize ADM involvement in AML pathogenesis, we investigated its expression during human hematopoiesis and in leukemic subsets, based on a morphological, cytogenetic and molecular characterization and in T cells from AML patients. In hematopoietic stem/progenitor cells and T lymphocytes from healthy subjects, *ADM* transcript was barely detectable. It was expressed at low levels by megakaryocytes and erythroblasts, while higher levels were measured in neutrophils, monocytes and plasma cells. Moreover, cells populating the hematopoietic niche, including mesenchymal stem cells, showed to express *ADM*. *ADM* was overexpressed in AML cells *versus* normal CD34^+^ cells and in the subset of leukemia compared with hematopoietic stem cells. In parallel, we detected a significant variation of *ADM* expression among cytogenetic subgroups, measuring the highest levels in inv(16)/t(16;16) or complex karyotype AML. According to the mutational status of AML-related genes, the analysis showed a lower expression of *ADM* in *FLT3*-ITD, *NPM1*-mutated AML and *FLT3*-ITD/*NPM1*-mutated cases compared with wild-type ones. Moreover, *ADM* expression had a negative impact on overall survival within the favorable risk class, while showing a potential positive impact within the subgroup receiving a not-intensive treatment. The expression of 135 genes involved in leukemogenesis, regulation of cell proliferation, ferroptosis, protection from apoptosis, HIF-1α signaling, JAK-STAT pathway, immune and inflammatory responses was correlated with *ADM* levels in the bone marrow cells of at least two AML cohorts. Moreover, *ADM* was upregulated in CD4^+^ T and CD8^+^ T cells from AML patients compared with healthy controls and some *ADM* co-expressed genes participate in a signature of immune tolerance that characterizes CD4^+^ T cells from leukemic patients. Overall, our study shows that *ADM* expression in AML associates with a stem cell phenotype, inflammatory signatures and genes related to immunosuppression, all factors that contribute to therapy resistance and disease relapse.

## Introduction

Adrenomedullin (ADM) is a 52-amino acid hormone belonging to the amylin/calcitonin gene-related peptide (CGRP) super-family, that has been originally identified in the extracts of human pheochromocytoma (1). It is produced by cleavage of an immature precursor that is synthesized by the *ADM* gene. ADM binds to calcitonin receptor-like (CALCRL), associated with modulating proteins with a single transmembrane domain, named receptor activity-modifying protein 2 (RAMP-2) or RAMP-3 (2).

ADM has been detected in many human tissues, including the endothelium, the nervous, cardiovascular, digestive, excretory, respiratory systems, the endocrine and reproductive organs (3). Despite its original definition as a hypotensive and vasodilator agent (1, 4), ADM is involved in a number of physiological processes, including angiogenesis (5), cell proliferation, migration (6), apoptosis (7, 8) and differentiation (9), with potential promoting and inhibitory functions depending on the cell type. Moreover, ADM production increases during infection, since it acts as an anti-microbial peptide against Gram-positive and Gram-negative bacteria (10), and during inflammation. Indeed, inflammatory molecules, as lipopolysaccharide (LPS) and 12-O-Tetradecanoylphorbol-13-acetate (TPA), and cytokines, including TNF-α and IL-1α force ADM secretion (11), and NF-κB binding sites have been identified on the *ADM* promoter (12). Once released, ADM can exert local and systemic anti-inflammatory actions by regulating cytokine secretion (13) and immune system properties, with beneficial effects on inflammatory conditions as gastric ulcers (14) and bowel diseases (15). Additional stimuli, as cell-to-cell interaction (16), growth factors, steroids, hormones, and physical factors, including oxidative stress and hypoxia, can induce *ADM* expression (3).

In the hematopoietic system, ADM is produced and secreted by peripheral blood monocytes, monocyte-derived macrophages and granulocytes (17). Moreover, mononuclear hematopoietic cells of the cord blood express *ADM* transcript (18) and ADM, in combination with growth-promoting cytokines, was able to enhance clonal growth and expansion of cord blood hematopoietic stem cells (18–20) and progenitor cells, respectively (20).

In contrast with the protective and therapeutic activity demonstrated in different diseases, ADM has pro-tumorigenic functions. It is over-expressed in a number of malignancies, including breast cancer, melanoma, tumors of the eye, of the respiratory, nervous, urogenital and gastroenteric system (21). Despite its relevance in hematopoietic stem cells and in the myeloid lineage, little is known about ADM in acute myeloid leukemia (AML). Previous studies on AML cellular models showed that HL60 (22) and THP1 cells produce ADM, though at low levels, and respond to a number of stimuli, including TPA, LPS, TNF-α by increasing its expression (17, 23). The elevated ADM production was associated with increased expression of markers of monocyte/macrophage differentiation (17). Conversely, exposure to exogenous ADM had no evident effects on cell differentiation that was instead induced by treatment with an ADM receptor antagonist. Exogenous ADM promoted AML cell proliferation through the ERK/MAPK pathway and induced CD31 upregulation, which could enhance their transendothelial migration capacity.

Here we analyzed the expression of the *ADM* gene across human hematopoietic cell differentiation and in AML, including its morphological, cytogenetic and molecular subtypes, cell subpopulations and T cell subsets from leukemic patients, and we investigated *ADM* impact on prognosis and its related transcriptional network.

## Materials and Methods

### Sample Collection and Cell Preparation

Samples were obtained from AML and acute lymphoblastic leukemia (ALL) patients after written informed consent, as approved by the institutional ethics committees (Comitato Etico Indipendente di Area Vasta Emilia Centro, protocol 112/2014/U/Tess and Comitato Etico della Romagna, protocol 5805/2019), in accordance with the Declaration of Helsinki. Mononuclear cells from bone marrow (n = 7) or peripheral blood (n = 5) of adult (non-M3) AML patients at diagnosis were collected by density gradient centrifugation using Lymphosep (Biowest). CD34^+^ leukemic blasts were enriched by immunomagnetic separation (CD34 MicroBead Kit, Miltenyi Biotec). Healthy hematopoietic stem-progenitor cells (CD34^+^) from bone marrow specimens (n = 3) were obtained by STEMCELL Technologies Inc.

### Gene Expression Datasets

Gene expression data were obtained from the BLUEPRINT consortium (http://dcc.blueprint-epigenome.eu/#/home) (24) and the Gene Expression Omnibus (GEO) repository [https://www.ncbi.nlm.nih.gov/gds, GSE98791 (25), GSE24759 (26), GSE24006 (27), GSE63270 (28), GSE158596 (29), GSE117090 (30), GSE14924 (31), GSE14468 (32), GSE6891 (33), GSE13159 (34)]. Array data from 61 AML bone marrow samples (blasts ≥80%) and 29 Philadelphia-negative (Ph−) B-ALL have been generated by the Next Generation Sequencing platform for targeted Personalized Therapy of Leukemia (NGS-PTL) project, as previously described (35, 36). The Beat AML (37) and The Cancer Genome Atlas (TCGA) project on AML (38) transcriptomic cohorts were obtained from https://portal.gdc.cancer.gov (projects BEATAML1.0-COHORT and TCGA-LAML), respectively. The datasets used in the manuscript are described in **Supplementary Table 1**.

### Transcriptomic Data Analysis

Data quality control and normalization (signal space transformation robust multi-array average) of NGS-PTL data (Affymetrix Human Transcriptome Array 2.0) were carried out by Expression Console software (version 1.4.1, Affymetrix, Thermo Fisher Scientific). Raw data from GSE24006, GSE14468, GSE6891, GSE13159 (all Affymetrix U133 Plus 2.0 array), were normalized by Transcriptome Analysis Console Software (version 4.0.1) using robust multi-array average normalization. Normalized data from GSE98791 (Agilent-021441 NCode Human Long Non-coding RNA), GSE117090 (Affymetrix Human Transcriptome Array 2.0), GSE14924, GSE63270 (Affymetrix U133 Plus 2.0 array) and GSE24759 (Affymetrix HT-HG-U133A Early Access) were retrieved from GEO. BLUEPRINT RNA-seq data were normalized in Transcript Per Million (TPM) by RSEM (39). RNA-Seq data from TCGA-LAML and Beat AML are available in the form of HTSeq read counts. Those were transformed into Counts Per Million (CPM) with Trimmed Mean of M values (TMM), using calcNormFactors (method = “TMM”) function in edgeR (40) (v3.24.1), then log2-transformed. RNA-seq data from GSE158596 were normalized using the median of ratios method of DESeq2 (41). Supervised gene expression analysis was performed by Student’s t-test or Welch’s t-test [R package stats, v3.4.1 (42)—python v3.6.5 (43) package scipy v1.5.2 (44)] in order to compare expression of *ADM*, its co-expressed and interacting genes. *ADM* interacting proteins were identified by STRING (version 11.0). Pathway enrichment analysis was carried out by Enrich R (45) on Gene Ontology Biological Processes, KEGG and Reactome annotations. The ClueGO package (version 2.5.7) from the Cytoscape software platform (version 3.8.1) was used for functional network analysis. Gene set enrichment analysis (GSEA) was performed with GSEA software (Broad Institute) (46).

### qRT-PCR

After TRIzol extraction, RNA was reverse transcribed into cDNA (PrimeScript Reag Kit with gDNA Eraser, Takara). TaqMan gene expression for *ADM* mRNA (Hs00181605_m1, ThermoFisher Scientific) was performed on CD34^+^ cells from AML and control samples, using *HPRT1* (Hs02800695_m1) as reference gene, on the Applied Biosystems 7500 Real-Time PCR System (ThermoFisher Scientific). Gene expression was quantified by the 2^−ΔΔCt^ method, using the average expression of healthy CD34^+^ cells as calibrator.

### Statistical Analyses

Data were reported as median and minimum-to-maximum values for continuous variables and as natural frequencies and percentages for categorical ones. The Shapiro–Wilk test was used to assess if continuous variables were normally distributed. The association between one continuous and one categorical variable was performed using the Student t-test or the Analysis of Variance (ANOVA) or the analogous Wilcoxon–Mann–Whitney test or Kruskal–Wallis test, as appropriate. In case of a significant result (*p*-value ≤0.05) from an omnibus test for the comparison of more than two categories, post-hoc test p-values were adjusted using the Bonferroni method. The association between two categorical variables was assessed by means of the Chi-square test or the Fisher’s exact test, as appropriate. Correlation among genes was studied through the Pearson correlation coefficient. To investigate the association between *ADM* expression and overall survival (OS), a Cox proportional hazards model was used. Hazard ratios (HRs) and 95% confidence intervals (CIs) were reported. To assess the presence of outliers or influential observations as well as the functional form of *ADM* in relation to the hazard function, the Deviance and the Martingale residuals were used, respectively. Overall survival analysis was performed firstly on each cohort separately and then on an integrated dataset to explore the prognostic role of *ADM* in specific subgroups otherwise characterized by very low frequency. Such integrated dataset was obtained applying the Blom transformation to the normalized expression data of *ADM* (47). Such rank-based transformation backtransforms the uniformly distributed ranks to a standard normal distribution. Statistical analyses were performed using R statistical language version 3.6.1 and STATA 12.0 (College Station).

## Results

### *ADM* Expression Is a Characteristic of the Myeloid Differentiation Program

**Figure 1:**
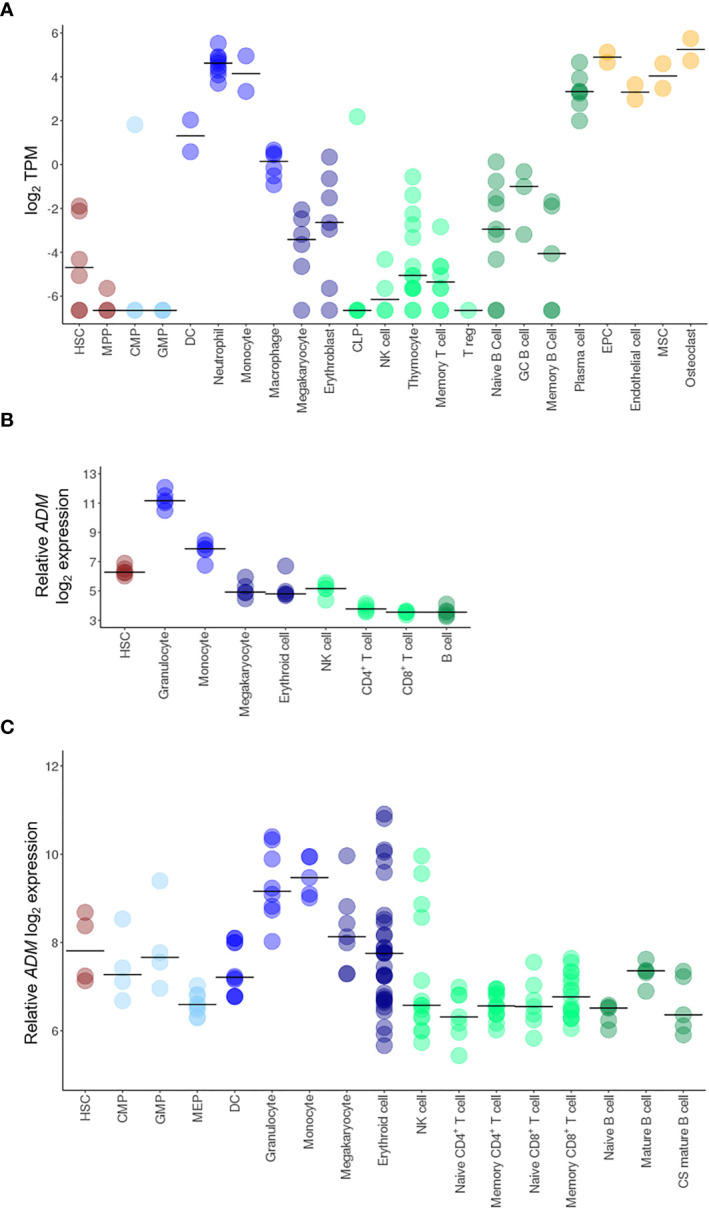
*ADM* expression in HSC and in the hematopoietic system. Transcriptional analysis of *ADM* expression in hematopoietic cells from the BLUEPRINT **(A)**, the GSE98791 **(B)** and the GSE24759 **(C)** datasets. Scatter plots were generated with the R package ggplot2 (48) (version 3.3.1). Each dot indicates one sample and the bar represents the median value (HSC, hematopoietic stem cells; MPP, hematopoietic multipotent progenitor cells; CMP, common myeloid progenitors; GMP, granulocyte–monocyte progenitors; DC, conventional dendritic cells, CLP, common lymphoid progenitors; NK, natural killer; T reg, regulatory T cells; GC, germinal center; EPC, endothelial progenitor cells; MSC, mesenchymal stem cells; MEP, megakaryocyte-erythroid progenitors; CS, class-switched; TPM, Transcripts Per Million).

To deeply investigate *ADM* expression in the hematopoietic system, we analyzed its mRNA levels at different stages of hematopoiesis and in the microenvironment in the BLUEPRINT dataset. *ADM* transcript was barely detectable in hematopoietic stem cells (HSC) and almost undetectable in hematopoietic multipotent progenitor cells (MPP) (**Figure 1A**). In the myeloid differentiation program, *ADM* was not expressed by common myeloid progenitor cells (CMP) and granulocyte-monocyte progenitor cells (GMP). It was barely detectable in megakaryocytes and erythroblasts, while increasing in dendritic cells (DC) and, especially, during neutrophilic differentiation and in monocytes (**Figure 1A**). Monocyte-derived macrophages showed low *ADM* levels. However, *ADM* expression was significantly enhanced by LPS stimulation (**Supplementary Figure 1**), in line with previous reports (49). In the lymphoid lineage, *ADM* was not expressed by common lymphoid progenitors (CLP) and by T cells at any stage of differentiation (thymocytes, memory T cells and regulatory T cells, **Figure 1A**). *ADM* expression remained close to undetectable during B lymphocyte differentiation (naïve, germinal center and memory B cells), while increasing in terminally-differentiated plasma cells (**Figure 1A**). Of note, *ADM* was highly expressed by cells populating the hematopoietic niche and/or interacting with the hematopoietic system, including endothelial progenitor and mature cells, bone marrow mesenchymal stem cells (MSC) and osteoclasts (**Figure 1A**).

To validate these data in independent cohorts, we analyzed the GSE98791 and GSE24759 datasets, containing hematopoietic and immune cell populations. The results were largely overlapping, showing very low *ADM* levels in CMP, GMP, B cells, CD4^+^ and CD8^+^ T lymphocytes (naïve, mature and memory), low expression in HSC, erythroid cells and megakaryocytes and higher *ADM* transcript in monocytes and granulocytes (**Figures 1B, C**).

Taken together, these data indicate that in the normal hematopoietic system, *ADM* expression is a hallmark of mature myeloid cells.

### Leukemia Stem Cells Express *ADM*

It was previously reported that the expression of the ADM binding receptor CALCRL is a prognostic marker in AML (50). However, both CGRP and ADM bind to the same receptor. To understand whether ADM may be involved in CALCRL signaling in AML, we analyzed its expression in three different datasets of leukemic and hematopoietic cells at different stages of differentiation, including stem and progenitor cells, defined on a surface phenotype base (GSE24006, GSE117190 and GSE63270).

**Figure 2:**
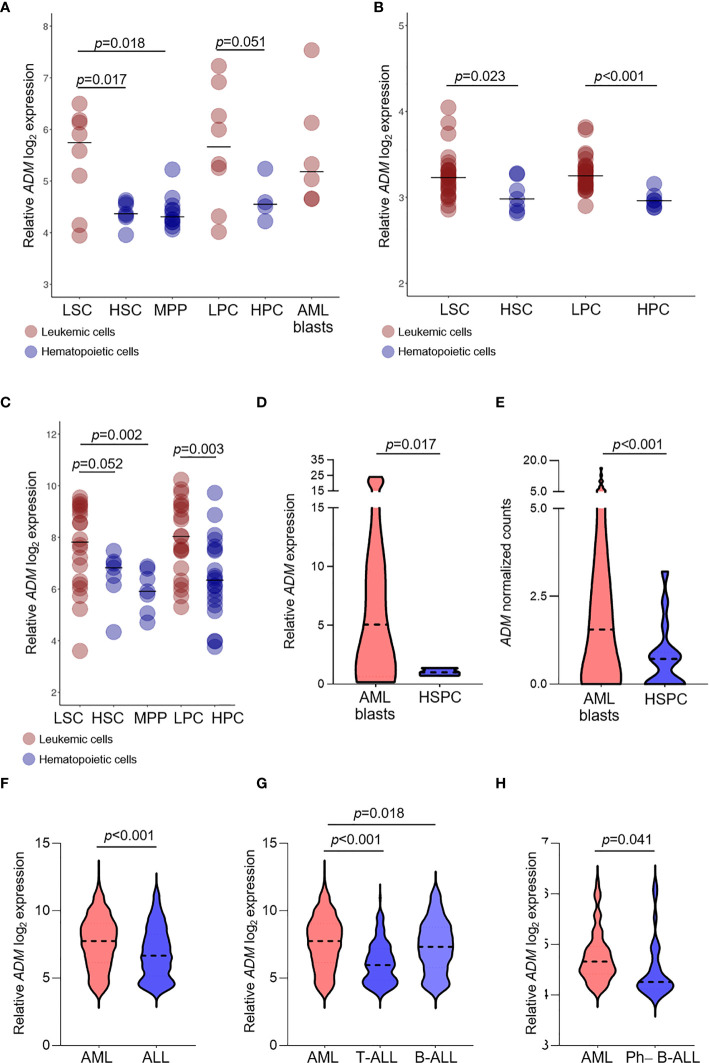
*ADM* expression is elevated in leukemic compared with hematopoietic stem and progenitor cells and in AML compared with ALL. **(A–C)** Comparison of *ADM* levels between leukemic cells subpopulations and normal stem and progenitor cells in the GSE24006 **(A)**, the GSE117090 **(B)** and the GSE63270 **(C)** datasets. Subpopulations according to their surface phenotype: leukemia stem cells (LSC): (Lin^−^)CD34^+^CD38^−^(CD90^−^), hematopoietic stem cells (HSC): Lin^−^CD34^+^CD38^−^CD90^+^(CD45RA^+/−^), hematopoietic multipotent progenitor cells (MPP): Lin^−^CD34^+^CD38^−^CD90^−^CD45RA^−^, leukemia progenitor cells (LPC): (Lin^−^)CD34^+^CD38^+^(CD90^−^), hematopoietic progenitor cells (HPC): Lin^−^CD34^+^CD38^+^(CD90^+^), AML blasts: Lin^−^CD34^−^. Scatter plots were generated with the R package ggplot2 (48) (version 3.3.1). Each dot indicates one sample and the bar represents the median value. **(D)** Comparison of *ADM* levels between AML blasts (n = 12) and healthy CD34^+^ bone marrow cells (n = 3, hematopoietic stem-progenitor cells, HSPC, qRT-PCR) and **(E)** between AML blasts (n = 60) and healthy G-CSF mobilized HSPC (n = 16) from the GSE158596 dataset. **(F)** *ADM* transcript levels in AML (n = 505) and ALL (n = 784, GSE13159), **(G)** separated in T-ALL (n = 173) and B-ALL (n = 441) and **(H)** in AML (n = 61) *versus* Ph−B-ALL (n = 29, NGS-PTL). Violin plots were generated with GraphPad Prims (version 8.4.3). The plots represent the frequency distribution of *ADM* levels (from minimum to maximum) and the dotted line indicates the median value.

*ADM* was overexpressed in leukemic stem cells (LSC) compared with HSC (GSE24006, *p* = 0.017; GSE117190, *p* = 0.023, GSE63270, *p* = 0.052) or MPP (GSE24006, *p* = 0.018; GSE63270, *p* = 0.002, **Figures 2A–C**). Elevated levels were also detected in leukemic compared with hematopoietic progenitor cells (GSE24006, *p* = 0.051; GSE117190, *p <*0.001, GSE63270, *p* = 0.003; **Figures 2A–C**). Moreover, *ADM* expression was maintained in AML blast cells that showed similar levels compared with more undifferentiated AML cells (GSE24006, **Figure 2A**). The elevated *ADM* expression in AML was confirmed in our qRT-PCR analysis of CD34^+^ AML cells *versus* bone marrow hematopoietic stem-progenitor cells (HSPC, *p* = 0.017, **Figure 2D**) and in the GSE158596 dataset, by comparing leukemic blasts with G-CSF mobilized HSPC (*p <*0.001, **Figure 2E**).

We then asked whether *ADM* expression differs between AML and ALL. We observed higher *ADM* expression in AML *versus* ALL (GSE13159, *p <*0.001, **Figure 2F**). This result was confirmed by comparing AML with T-ALL (*p <*0.001, **Figure 2G**) or B-ALL (*p* = 0.018, **Figure 2G**), separately. When analyzing the NGS-PTL B-ALL cohort, we observed that *ADM* expression was increased in AML compared with Ph-negative (Ph−) B-ALL (NGS-PTL, *p* = 0.041, **Figure 2H**). Overall, these data suggest that *ADM* is generally higher in AML compared with ALL.

### High *ADM* Expression Associates With Specific Cytogenetic Features in AML

We then asked whether *ADM* expression levels changed among AML molecular and biological subgroups and investigated the association between *ADM* levels and disease features, including age, morphology, cytogenetics, and genomic lesions. To this aim, we analyzed six independent datasets of newly-diagnosed AML (excluding M3 cases, age ≥18 years) and focused on bone marrow samples (GSE6891, n = 68; Beat AML, n = 142; TCGA-LAML, n = 135, GSE13159, n = 458, NGS-PTL, n = 61), except for GSE14468 cohort that included mixed bone marrow and peripheral blood samples (n = 459, tissue not specified, **Table 1** and **Supplementary Table 2**).

**Table 1:** Association between *ADM* expression levels and clinical/molecular data across public datasets.

Variable	GSE6891 (n=68)			GSE14468 (n=459)			Beat AML (n=142)			TCGA-LAML (n=135)			
	n (%)	median [min-max]	*p*	n (%)	median [min-max]	*p*	n (%)	median [min-max]	*p*		n (%)	median [min-max]	*p*
**Age**			**-**			**0.034**			0.653				0.056
<60-years	–	–		383 (83.8)	159.8 [57.7-3848.3]		60 (42.3)	9.5 [0.2-313.9]			72 (53.3)	1.3 [0.0-23.1]	
≥60-years	63 (100.0)	196.7 [93.4-4451.3]		74 (16.2)	238.2 [98.4-442.9]		82 (57.7)	11.4 [0.3-151.7]			63 (46.7)	2.0 [0.0-57.1]	
*missing*	5			2			–				–		
**FAB**			**0.001**			**<0.001**			0.057				0.591
M0	2 (3.6)	229.7 [188.7-270.6]		18 (4.1)	135.3 [80.5-477.7]		4 (7.8)	4.0 [1.2-6.1]			14 (10.5)	2.1 [0.0-5.5]	
M1	11 (19.6)	122.7 [97.0-206.5]		97 (22.2)	134.8 [57.7-3396.9]		6 (11.8)	1.3 [1.0-118.2]			35 (26.1)	1.2 [0.0-12.4]	
M2	23 (41.1)	148.1 [92.4-1024.0]		123 (28.2)	147.0 [71.0-2936.7]		5 (9.8)	6.4 [0.9-63.6]			38 (28.4)	1.3 [0.1-17.2]	
M4	9 (16.1)	221.3 [172.4-4451.3]		83 (19.0)	205.1 [84.5-3848.3]		16 (31.4)	19.0 [3.5-159.1]			29 (21.6)	2.0 [0.1-12.8]	
M5	10 (17.9)	448.6 [166.6-4451.3]		109 (24.9)	183.6 [83.9-4482.2]		19 (37.3)	4.3 [0.3-151.7]			15 (11.2)	1.1 [0.0-57.1]	
M6	1 (1.8)	843.4		7 (1.6)	171.3 [123.6-849.2]		–	–			2 (1.5)	2.3 [1.9-2.7]	
M7	–	–		–	–		1 (2.0)	5.9			1 (0.8)	1.4 [1.4-1.4]	
*missing*	12			22			91				1		
**Cytogenetic group**			0.063			**<0.001**			**0.001**				**0.001**
t(8;21)	3 (5.0)	104.0 [96.3-126.2]		32 (9.8)	137.2 [92.4-451.9]		3 (2.1)	7.3 [0.9-15.1]			7 (5.3)	0.2 [0.1-1.0]	
inv(16)/t(16;16)	4 (6.7)	516.6 [182.3-2256.7]		33 (10.1)	210.1 [125.4-2164.8]		9 (6.4)	18.9 [5.1-98.7]			10 (7.6)	1.9 [1.2-7.0]	
NK	23 (38.3)	188.7 [96.3-4451.3]		139 (42.4)	149.1 [57.7-3848.3]		75 (53.6)	7.5 [0.2-313.9]			60 (45.5)	1.2 [0.0-57.1]	
CK	4 (6.7)	178.1 [92.4-4451.3]		27 (8.2)	133.4 [95.7-2740.1]		19 (13.6)	35.6 [1.2-130.8]			18 (13.6)	3.4 [0.3-17.2]	
*KMT2A*-r	1 (1.7)	179.8		15 (4.6)	116.2 [84.5-1782.9]		8 (5.7)	2.0 [0.3-39.3]			8 (6.1)	0.5 [0.0-1.8]	
Other	25 (41.7)	229.1 [114.6-2019.8]		82 (25.0)	169.5 [74.5-3304.0]		26 (18.6)	13.8 [0.4-159.1]			29 (22.0)	1.9 [0.0-12.8]	
*missing*	8			131			2				3		
***FLT3*-ITD**			**0.023**			**0.009**			0.062				**0.001**
*FLT3*-ITD^+^	18 (26.5)	145.5[96.3-1024.0]		112 (28.3)	144.5 [57.7-2091.0]		27 (20.3)	4.2 [0.4-118.2]			27 (20.5)	0.4 [0.0-23.1]	
*FLT3*-ITD**^−^**	50 (73.5)	247.3 [92.4-4451.3]		283 (71.7)	167.7 [68.1-3848.3]		106 (79.7)	14.5 [0.2-313.9]			105 (79.6)	1.9 [0.0-57.1]	
*missing*	–			64			9				3		
***NPM1* status**			0.127			0.249			0.064				**0.034**
*NPM1*-mut	20 (29.4)	151.2 [96.3-2019.8]		135 (34.2)	313.9 [57.7-3743.1]		37 (27.8)	4.2 [0.6-313.9]			38 (28.8)	0.6 [0.0-57.1]	
*NPM1*-wt	48 (70.6)	244.0 [92.4-4451.3]		260 (65.8)	164.9 [68.1-3848.3]		96 (72.2)	13.8 [0.2-159.1]			94 (71.2)	1.8 [0.0-16.8]	
*missing*	–			64			9				3		
***FLT3*-ITD/*NPM1***			**0.004**			0.070			0.086				**0.009**
−/wt	40 (58.8)	244.7 [92.4-4451.3]		218 (55.2)	168.9 [68.1-3848.3]		81 (60.9)	15.1 [0.2-159.1]			84 (63.6)	2.0 [0.0-16.8]	
+/wt	8 (11.8)	281.9 [97.0-1024.0]		42 (10.6)	147.5 [83.3-2091.0]		15 (11.3)	8.9 [0.4-118.2]			10 (7.6)	0.8 [0.2-12.4]	
−/mut	10 (14.7)	305.0 [120.3-2019.8]		65 (16.5)	163.2 [71.0-3743.1]		25 (18.8)	9.4 [0.6-313.9]			21 (15.9)	1.5 [0.0-57.1]	
+/mut	10 (14.7)	123.8 [96.3-166.6]		70 (17.7)	140.6 [57.7-1758.3]		12 (9.0)	2.7 [0.8-46.5]			17 (12.9)	0.3 [0.0-23.1]	
*missing*	–			64			9				3		
**ELN 2010**			0.448			0.255			0.104				0.114
Favorable	11 (18.3)	221.3 [96.3-2256.7]		115 (35.3)	172.4 [67.2-2179.8]		38 (27.1)	10.8 [0.5-313.9]			40 (30.8)	1.3 [0.0-57.1]	
Int-I	19 (31.7)	148.1 [96.3-4451.3]		89 (27.3)	140.1 [57.3-3875.1]		49 (35.0)	7.7 [0.2-151.7]			37 (28.5)	1.2 [0.1-23.1]	
Int-II	18 (30.0)	201.6 [117.8-2019.8]		74 (22.7)	160.9 [70.0-3327.0]		26 (18.6)	10.2 [0.3-159.1]			22 (16.9)	1.5 [0.0-12.8]	
Adverse	12 (20.0)	237.3 [92.4-4451.3]		48 (14.7)	150.1 [83.7-2019.8]		27 (19.3)	21.8 [0.4-130.8]			31 (23.9)	2.8 [0.3-17.2]	
*missing*	8			133			2				5		

CK, complex karyotype; Int, Intermediate; ITD, internal tandem duplication; KMT2A-r, KMT2A-rearranged; min-max, minimum-to-maximum value; mut, mutated; n, number; NK, normal karyotype; p, p value; wt, wild-type.

p ≤ 0.05 are highlighted as bold text.

Elderly patients (aged ≥60 years) expressed significantly higher *ADM* transcript in the GSE14468 cohort (*p* = 0.034) and this comparison was close to significance in the TCGA-LAML dataset (*p* = 0.056). *ADM* expression showed a significant variation according to French–American–British classification (GSE6891, *p* = 0.001, GSE14468, *p <*0.001), with high levels in the immature M0 cytomorphology, in the monocytic types (FAB M4/M5) and in erythroid leukemia (M6). Moreover, we observed a significant variation among cytogenetic subgroups (Beat AML, *p* = 0.001; TCGA-LAML, *p* = 0.001, GSE14468, *p <*0.001; GSE13159, *p <*0.001), with elevated levels in complex karyotype and inv(16)/t(16;16) AML and low expression in t(8;21) cases.

The analysis of *ADM* expression according to genetic alteration of AML-related genes revealed no association with the mutational status of *IDH1*, *IDH2*, *KRAS*/*NRAS*, *RUNX1*, *ASXL1*, *DNMT3A* and *TP53*. Moreover, no significant differences were observed among ELN 2010 risk categories (**Table 1**). Conversely, we detected lower expression in *FLT3*-ITD AML (GSE6891, *p* = 0.023; TCGA-LAML, *p* = 0.001; GSE144468, *p* = 0.009) compared with *FLT3*-ITD-negative AML and in *NPM1*-mutated *versus* wild-type cases in the TCGA-LAML dataset (*p* = 0.034). Accordingly, when considering *FLT3*-ITD and *NPM1* mutation simultaneously, we observed a significant difference among the subgroups, with the wild-type cases expressing the highest *ADM* levels and the *FLT3*-ITD/*NPM1*-mutated ones expressing the lowest ones (GSE6891, *p* = 0.004; TCGA-LAML, *p* = 0.009).

**Figure 3:**
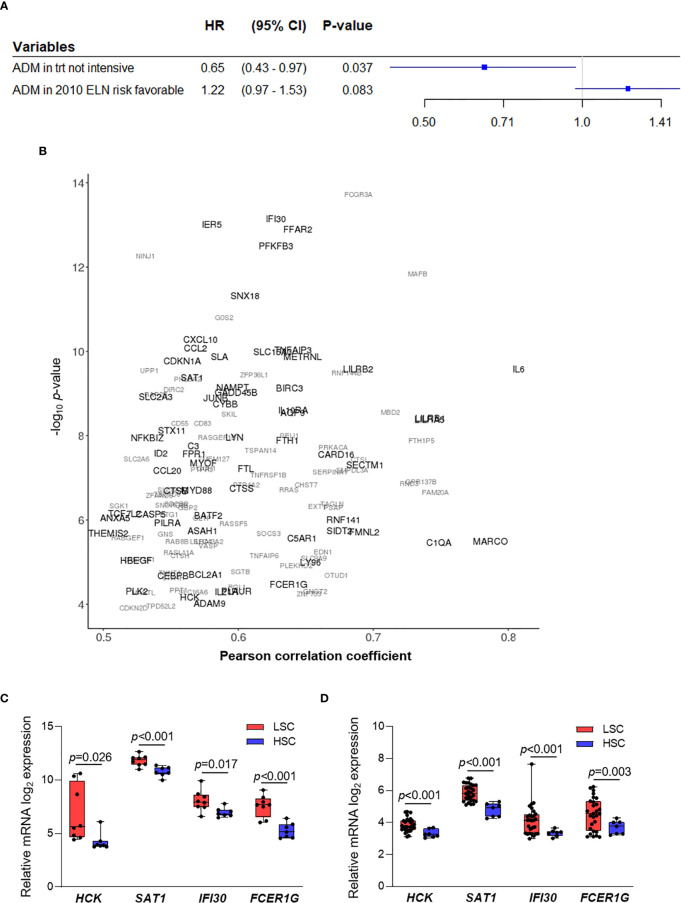
*ADM* prognostic role and co-expressed genes in AML. **(A)** Results from two separate Cox regression models within the subgroup with 2010 ELN favorable risk (adjusting for age, n = 214) and within the subgroup receiving a not-intensive treatment (n = 64, HR, Hazard ratio, CI, confidence interval, trt, treatment). **(B)** Correlation analysis between *ADM* expression and the AML transcriptome across bone marrow samples from five AML datasets (GSE6891, GSE13159, Beat AML, TCGA-LAML, NGS-PTL). Genes showing an absolute value of Pearson correlation coefficient ≥0.50 and a *p* value ≤0.05 in at least two cohorts were reported. Genes are represented according to the weighted arithmetic mean of the correlation coefficient and *p* value across the datasets. The scatter plot was generated with the R package ggplot2 (48) (version 3.3.1). **(C)** Transcriptional analysis of *ADM* co-expressed genes in LSC compared with HSC in the GSE24006 and **(D)** in the GSE117090 datasets (fold change ≥1.5 and *p ≤*0.05 were set as cut off). The boxes extend from minimum to maximum values, each individual value is plotted and the line represents the median value.

We then asked whether *ADM* may have a prognostic role in terms of OS, but no statistically significant association was observed. This analysis was performed on the Beat AML, TCGA-LAML, GSE6891 and NGS-PTL cohorts that had the information related to the OS. Bone marrow and peripheral blood samples were included in order to increase the cohort size and allow subgroup analyses. Considering the integrated dataset, the associations between *ADM* and AML molecular and biological features observed on the single cohorts were confirmed (**Table 2**). Moreover, the integrated dataset suggested the association with ELN 2010 risk classification (*p* = 0.006, **Table 2**). In details, *ADM* expression was elevated in the favorable and adverse risk categories, compared with the intermediate ones, in line with the cytogenetic features. Additional analyses showed that high *ADM* expression had a negative impact on OS within the patients’ subgroup characterized by favorable ELN 2010 risk (HR for a one-unit increase in *ADM* = 1.28; 95% CI: 1.02–1.61, *p* = 0.031, **Figure 3A**). Adjusting for age (<60, ≥60 years) that resulted either significantly associated to *ADM* and to affect the OS, the HR for a one-unit increase in *ADM* was equal to 1.22 (95% CI: 0.97–1.53, *p* = 0.083). Conversely, high *ADM* expression seemed to have a positive impact on OS within the subgroup receiving a not-intensive treatment (azacytidine, decitabine, targeted therapies; HR = 0.65, 95% CI: 0.43–0.97 *p* = 0.037, **Figure 3A**). In this cohort, the prognostic role of *ADM* was independent from other biological and clinical factors.

**Table 2:** Association between *ADM* expression levels and clinical/molecular data in the overall AML cohort after normalization.

Variable	Beat AML+TCGA-LAML+GSE6891+NGS-PTL (n=903)		
	n (%)	median [min-max]	*p*
**Age**			**<0.001**
<60-years	578 (64.9)	-0.1 [-3.0-2.6]	
≥60-years	313 (35.1)	0.2 [-2.8-2.9]	
*missing*	12		
**FAB**			**<0.001**
M0	40 (6.0)	-0.4 [-2.2-1.1]	
M1	147 (21.9)	-0.4 [-3.0-2.4]	
M2	175 (26.1)	-0.2 [-2.6-2.2]	
M4	143 (21.3)	0.2 [-1.9-2.9]	
M5	154 (23.0)	0.02 [-2.3-2.9]	
M6	9 (1.3)	0.1 [-0.6-1.4]	
M7	3 (0.5)	-0.1 [-0.4-0.4]	
*missing*	232		
**Cytogenetic group**			**<0.001**
t(8;21)	50 (6.2)	-0.5 [-1.6-1.1]	
inv(16)/t(16;16)	68 (8.4)	0.3 [-0.6-2.3]	
NK	360 (44.4)	-0.2 [-3.0-2.9]	
CK	89 (11.0)	0.4 [-1.7-2.9]	
*KMT2A*-r	38 (4.7)	-0.9 [-2.8-1.9]	
Other	205 (25.3)	0.1 [-2.6-2.8]	
*missing*	93		
**FLT3-ITD**			**<0.001**
*FLT3*-ITD^+^	200 (24.3)	-0.4 [-3.0-2.3]	
*FLT3*-ITD^−^	622 (75.7)	0.1 [-2.8-2.9]	
*missing*	81		
***NPM1* status**			**<0.001**
*NPM1*-mut	241 (30.4)	-0.3 [-3.0-2.6]	
*NPM1*-wt	572 (69.6)	0.1 [-2.8-2.9]	
*missing*	81		
***FLT3*-ITD/*NPM1***			**<0.001**
−/wt	486 (59.1)	0.2 [-2.8-2.9]	
+/wt	86 (10.5)	-0.2 [-2.1-1.7]	
−/mut	136 (16.6)	-0.01 [-2.6-2.6]	
+/mut	114 (13.9)	-0.6 [-3.0-2.3]	
*missing*	81		
**ELN 2010**			**0.006**
Favorable	224 (30.1)	0.1 [-2.7-2.6]	
Int-I	218 (29.3)	-0.2 [-3.0-2.9]	
Int-II	162 (21.7)	0.02 [-2.6-2.3]	
Adverse	141 (18.9)	0.4 [-2.8-2.9]	
*missing*	158		

CK, complex karyotype; ITD, internal tandem duplication; KMT2A-r, KMT2A-rearranged; min–max, minimum-to-maximum value; mut, mutated; n, number; NK, normal karyotype; p, p value; wt, wild-type.

p ≤ 0.05 are highlighted as bold text.

### The *ADM* Gene Network Is Enriched of Inflammatory Signatures in Leukemic Cells and of Immunomodulatory Genes in T Cells From AML Patients

To understand the biological features associated with *ADM* expression in AML, we analyzed genes co-expressed and interacting with it. We defined 135 genes whose expression positively correlated with *ADM* (absolute value of Pearson correlation coefficient ≥0.5 and *p* value ≤0.05) in at least two out of the five cohorts of bone marrow samples (**Figure 3B** and **Supplementary Table 3**). Moreover, we identified the top-scoring *ADM* interactors (protein–protein interaction enrichment *p <*1.0e−16, **Supplementary Figure 2**), that are mainly involved in G protein-coupled receptor signaling.

Genes co-expressed with *ADM* were involved in regulation of cell growth and proliferation (e.g. *CDKN2D*, *SDCBP*, *BTG1*, *PTPRJ*, *SGK1*), ferroptosis (*FTH1*, *CYBB*, *SAT1*, *FTL*), protection from apoptosis (e.g. *HCK*, *RNF144B*, *BCL2A1*, *BIRC3*), HIF-1α signaling (e.g. *CDKN1A*, *EDN1*, *PFKFB3*, *CYBB*), JAK-STAT pathway (*SOCS3*, *IL6*, *CDKN1A*, *IL10RA*, *IL21R*) and response to stimuli, including lipids, LPS, cytokines and chemokines (**Supplementary Table 4**). Three of these genes were confirmed in four out of five cohorts, thus representing high-fidelity *ADM* co-expressed genes. They were the p53 transcriptional target *SAT1*, that is involved in polyamine metabolism and functions as a metabolic mediator of ferroptotic cell death (51), the BCL2 family member *BCL2A1*, that has been recently shown to confer resistance to Venetoclax treatment (52, 53), and *IER5*, that regulates LPC proliferation (54) (**Figure 3B** and **Supplementary Table 3**).

Four *ADM* co-expressed genes were also upregulated in LSC compared with HSC in two analyzed datasets (GSE24006, **Figure 3C** and GSE117090, **Figure 3D**). The Src family kinase HCK is strongly expressed in a significant proportion of AML patients (55), is known to be upregulated in leukemic compared to normal stem cells and is a potential therapeutic target (56, 57). Along with the metabolic mediator *SAT1*, an additional gene functions as regulator of cell homeostasis, namely the lysosomal thiol reductase *IFI30*, that facilitates degradation of unfolded proteins, thus controlling endoplasmic-reticulum stress. *IFI30* is also a predictor of response to combined Mitoxantrone, Etoposide, Cytarabine and the proteasome inhibitor Ixazomib in relapsed/refractory AML (58). Moreover, *FCER1G* is an immune regulator. Indeed, it is an adapter protein that transduces activation signals from various immunoreceptors and has been shown to prime T cells toward T-helper 2 and T-helper 17 cell subtypes (59).

**Figure 4:**
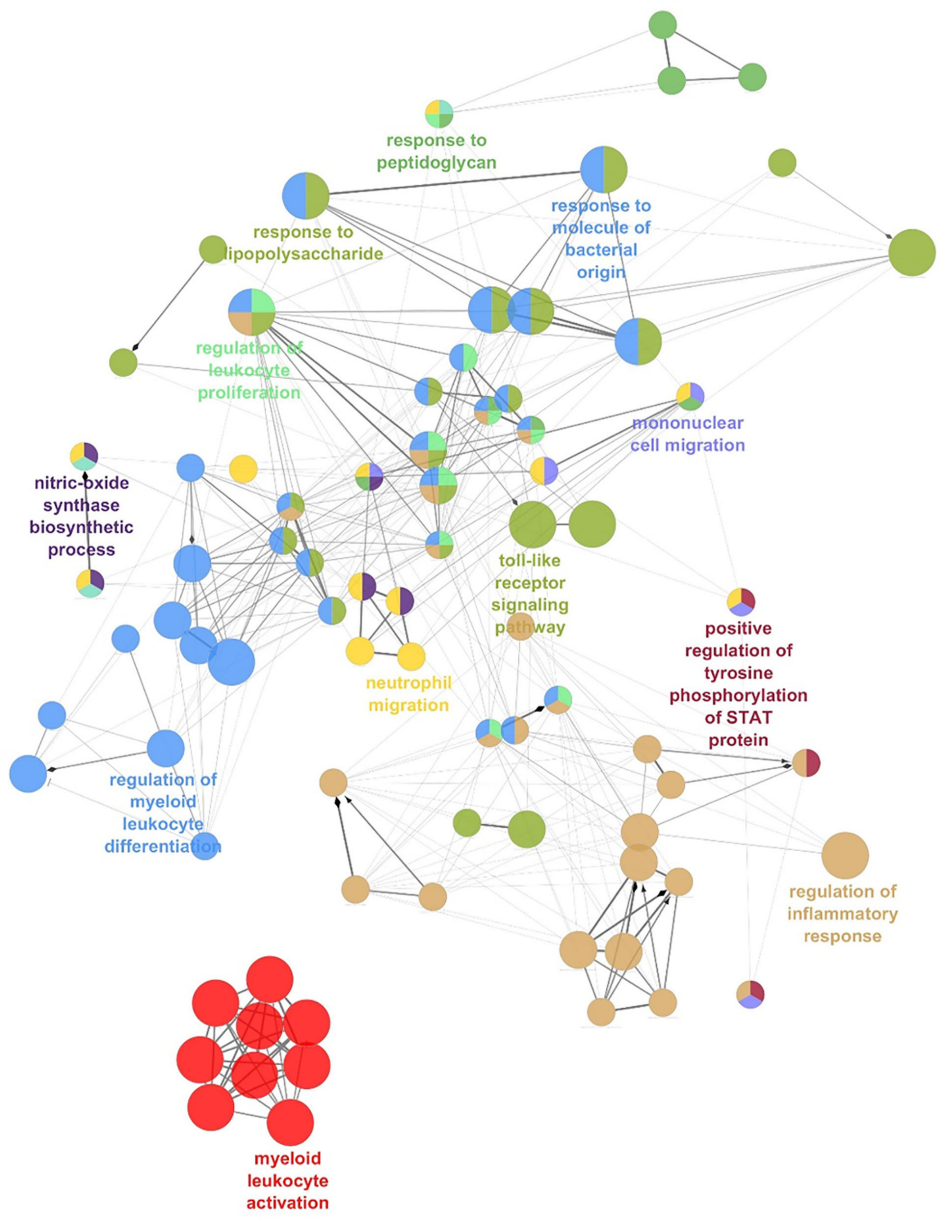
The network of *ADM* co-expressed genes in AML. Network analysis of the Gene Ontology Biological Processes pathways enriched by *ADM* co-expressed genes in AML (ClueGO). Colors indicate functionally-related pathways; one representative pathway for each subnetwork is specified.

Remarkably, network analysis of the *ADM* co-expressed genes across AML datasets highlighted the enrichment of transcripts involved in immune and inflammatory response, including myeloid leukocyte activation, regulation of their differentiation, neutrophil migration, toll-like receptor signaling, mononuclear cell migration, regulation of leukocyte proliferation (**Figure 4** and **Supplementary Table 4**), suggesting an association between high *ADM* levels and an inflammatory status in leukemic cells.

**Figure 5:**
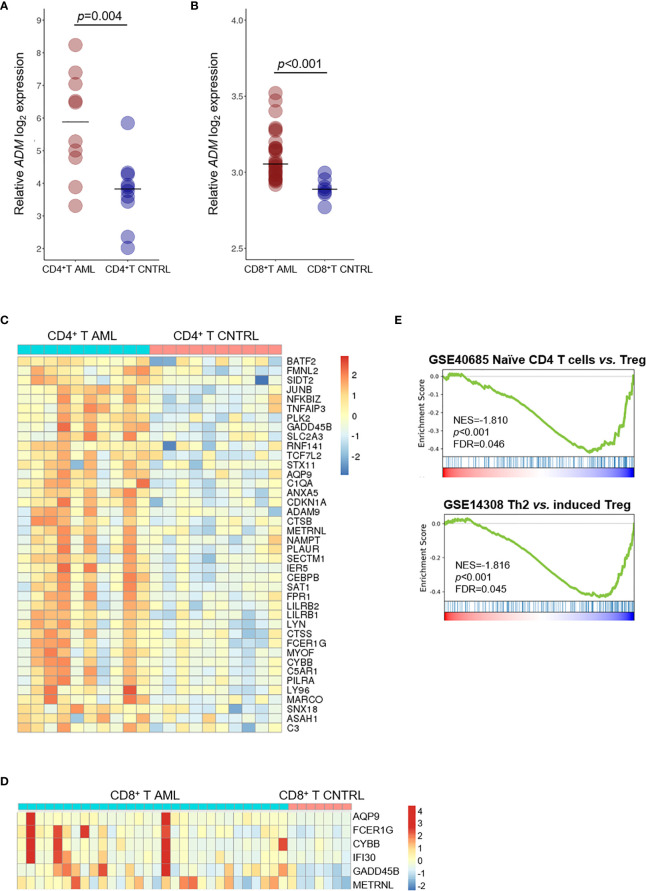
*ADM* is overexpressed in the T cell compartment of AML patients and associates with a tolerogenic gene signature. **(A)** Comparison of *ADM* levels between CD4^+^ T cells from AML patients and healthy subjects (GSE14924) and **(B)** between CD8^+^ T cells from AML patients and healthy subjects (GSE117090). Scatter plots were generated with the R package ggplot2 (48) (version 3.3.1). Each dot indicates one sample and the bar represents the median value (CNTRL: healthy subjects). **(C)** Heatmap of *ADM* co-expressed genes that are differentially expressed between CD4^+^ or **(D)** CD8^+^ T lymphocytes isolated from AML patients and from healthy subjects. Columns represent patients/subjects. Data were standardized through a z-score transformation. Color changes within a row indicate expression levels relative to the mean and rescaled on the transcript standard deviation. Genes are grouped according to average linkage hierarchical clustering. Heatmaps were built with R package pheatmap (64) (version 1.0.12). **(E)** GSEA of CD4^+^ T cells from AML patients compared with healthy subjects showing the enrichment of tolerance signatures (NES, normalized enrichment score, FDR, false discovery rate; Th2, T-helper 2, Treg, regulatory T cells).

Since *ADM* has been previously linked with immune response under physiological and pathological conditions (60–63), we compared its expression in T cell subsets from AML patients and healthy controls. We observed increased *ADM* expression in CD4^+^ T cells (GSE14924, *p <*0.001, **Figure 5A**) and in CD8^+^ T lymphocytes (GSE117090, *p <*0.001, **Figure 5B**) from AML patients. Moreover, 40 and six *ADM* co-expressed genes were upregulated in CD4^+^ T (**Figure 5C**) and CD8^+^ T cells (**Figure 5D**) from AML patients compared with cells from healthy controls, respectively. When studied by GSEA, CD4^+^ T cells from AML patients were enriched of signatures related to regulatory T (Treg) cells (**Figure 5E**). Of note, some *ADM* co-expressed genes, including *JUNB*, *CDKN1A*, *ANXA5*, *CYBB*, *NFKBIZ*, that were upregulated in CD4^+^ T cells from AML patients compared with cells from healthy controls (**Figure 5B**), are known to participate to the Treg phenotype (65–69).

## Discussion

ADM is a circulating hormone that also functions as a local paracrine and autocrine mediator, with involvement in a number of different cellular responses. We here studied *ADM* expression in the hematopoietic system and in AML and analyzed the transcriptional program associated with it both in leukemic cells and the immune microenvironment.

*ADM* is upregulated in a variety of human cancers compared with normal tissues and its mRNA expression correlated with high protein expression in the majority of them (21). In AML, we observed elevated levels in cell subpopulation defined, on the basis of their surface phenotype, as LSC and LPC compared with their normal counterparts that showed undetectable-to-barely-detectable levels.

*ADM* expression is also elevated in AML compared with ALL, in line with the observation that *ADM* expression is a main feature of the myeloid differentiation program. Accordingly, among AML FAB subtypes, monocytic (M4/M5) and erythroid (M6) leukemia had the highest expression, together with the immature M0 phenotype. These data reflect the distribution of *ADM* expression across the cytogenetic subgroups, with complex karyotypes, that frequently have an undifferentiated phenotype and inv(16)/t(16;16) AML, that characterizes the myelomonocytic cytomorphology, showing the strongest *ADM* positivity. This feature may also explain the lack of prognostic relevance of *ADM* in AML in general, that has been reported in other solid tumors (70–76). Conversely, high *ADM* levels showed a potential negative impact on overall survival in the favorable ELN 2010 risk class, that also includes inv(16)/t(16;16) cases. In AML, *ADM* expression is related to the disease molecular features, both in terms of genomic rearrangements and mutational status. Indeed, *FLT3*-ITD or *NPM1*-mutated cases displayed lower *ADM* levels compared with the wild-type ones and the expression was particularly low when the two alterations co-occurred. Several lines of evidence may explain this observation. First, two tyrosine kinases, namely *LYN* and *HCK*, show a positive correlation with *ADM*, suggesting alternative ways of signaling activation. Moreover, *ADM* is co-expressed with *SLAP*, that binds to FLT3 and modulates receptor stability and downstream signaling (77), likely favoring its activation even in the absence of the internal tandem duplication. Regarding *NPM1*-mutated AML, an inflammatory transcriptional program, characterized by enrichment of genes belonging to IFN-γ response, IL6 signaling and complement cascade (78) has been already associated with this genomic subgroup, and PRDM16 upregulation contributes to it (79). Functional studies on AML genomic subtypes will clarify in the future the role of ADM in specific leukemic cell contexts, thus overcoming the limitation of the currently available studies that analyzed generic AML models. Moreover, some of the identified associations and ADM function in AML cells under the pressure of not-intensive treatment regimens (e.g. hypomethylating agents), deserve further validation and investigation.

Our data suggest that the *ADM-*related transcriptional network has a role in cell proliferation, it negatively regulates apoptosis and, remarkably, it is involved in the inflammatory response. Moreover, some of the *ADM* co-expressed genes are already known for their leukemia-related role, including the anti-apoptotic gene *BIRC3*, the signaling molecules *HCK*, *LYN* (tyrosine kinases), *SLA* (Src kinase-like-adapter protein) and *PLAUR* (urokinase plasminogen activator surface receptor), the transcriptional regulators *TCF7L2* (WNT pathway) and *ID2*, the metabolism-related genes *FTH1*, *FTL* (ferritin heavy and light chain, respectively), *PFKFB3* (glycolytic regulator), *NAMPT* (NAD biosynthesis pathway), *SLC15A*3 (solute carriers transporting histidine) and *FFAR2* (free fatty acid receptor). Among *ADM* interacting genes, *EDN1*, that mediates VEGF-C-induced proliferation and chemoresistance in AML (80), showed a positive correlation with *ADM* expression. Several co-expressed genes and enriched pathways point towards an AML inflammatory phenotype, characterized by expression of *IL6*, *IL10RA*, *CXCL10*, *THEMIS2*, *TNFAIP3*, *LILRA5*, *LILRB2*. *ADM* was also upregulated in the CD4^+^ and CD8^+^ T cell subsets from AML patients compared with healthy controls and *ADM* correlating genes that were identified in AML, participate in a signature of immune tolerance in CD4^+^ T cells. We could not perform the correlation analysis on the CD4^+^ and CD8^+^ T cells due to the low number of samples, which would have hampered data significance and robustness.

In our analysis, none of the genes belonging to the ADM receptor complex (CALCRL, RAMP2, RAMP3) showed a correlation with endogenous *ADM* expression. However, it has been recently reported that CALCRL, the receptor of ADM and CGRP, is also overexpressed in LSC and its genomic ablation impaired the clonogenic capacity of AML cell lines (50) and the frequency of chemotherapy-resistant cells able to initiate leukemia relapse in preclinical models (81). In contrast to *ADM*, CALCRL was highly expressed in *NPM1*-mutated cases also carrying the *FLT3*-ITD (50). This difference reinforces the notion that the receptor expression is not controlled by the basal ADM levels, rather by changes in the extracellular ADM availability (22). We can therefore hypothesize an intrinsic capacity of leukemic cells to respond to ADM-mediated microenvironmental and self-stimulation, as also supported by the high level of *ADM* expression observed in MSC and osteoclasts.

Overall, our results suggest multiple biological roles of ADM in AML. Indeed, it may support LSC and may be involved in the maintenance of a leukemic cell inflammatory phenotype *via* autocrine and paracrine signaling, thus contributing to drug resistance and relapse. Moreover, ADM may exert an anti-inflammatory action when released in the blood and may promote immune tolerance by direct expression in the CD4^+^ T cell subset and by uptake from the tumor microenvironment, as indicated by data from murine models of autoimmune disorders (63). This evidence, along with the observation that an antagonistic ADM peptide induced differentiation of leukemic cell lines (22), suggest that targeting ADM may carry therapeutic potentials in AML. However, given the pleiotropic effects of ADM, a therapeutic strategy to deplete it may have serious side effects and toxicities. Therefore, encapsulated formulations aimed to deliver ADM neutralizing antibodies in targeted cells may be required. Alternatively, combination strategies blocking the *ADM*-related network (e.g. HCK, LYN, NAMPT inhibitors) may be investigated for their effect on AML cases expressing high *ADM* levels.

## Data Availability Statement

TThe NGS-PTL gene expression array AML dataset is available in the Gene Expression Omnibus repository under the accession number GSE161532.

## Ethics Statement

The studies involving human participants were reviewed and approved by Comitato Etico Indipendente di Area Vasta Emilia Centro (CE-AVEC), Bologna, Italy, and Comitato Etico della Romagna (CEROM), Meldola (FC), Italy. The patients provided their written informed consent to participate in this study.

## Author Contributions

GS designed the research, analyzed the data and wrote the manuscript. DA and EF analyzed transcriptomic data and prepared the figures. EP performed statistical analysis and revised the manuscript. SV and AS analyzed the data and revised the manuscript. AP, MGh, AF, VR, AA, and EO performed experiments. RL and MC helped in data interpretation. CP, CC, and MC provided patients’ specimens and data. GM supported the study design and data interpretation. MGo designed the research, analyzed the data and revised the manuscript. All authors contributed to the article and approved the submitted version.

## Funding

This study was supported by TrevisoAIL.

## Conflict of Interest

The authors declare that the research was conducted in the absence of any commercial or financial relationships that could be construed as a potential conflict of interest.
