# Supplementary material for: Adrenomedullin Expression Characterizes Leukemia Stem Cells and Associates With an Inflammatory Signature in Acute Myeloid Leukemia
Source: Front Oncol. 2021 Jun 2;11:684396. doi: 10.3389/fonc.2021.684396 (PMC8208888; doi:10.3389/fonc.2021.684396)
Supplement: Supplementary file 1 [file DataSheet_1.pdf]

Supplementary Material

Supplementary Figures

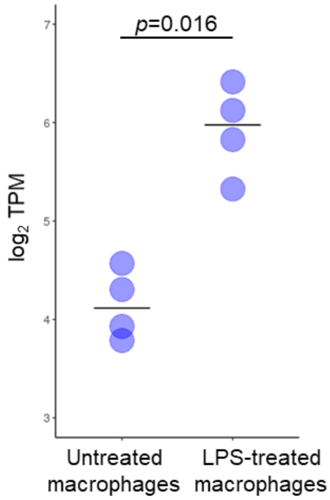

Supplementary Figure 1. LPS-induced upregulation of *ADM* in macrophages. Comparison of *ADM* levels between untreated and LPS-treated macrophages (day 6) from the BLEUPRINT dataset (TPM: Transcripts Per Million).

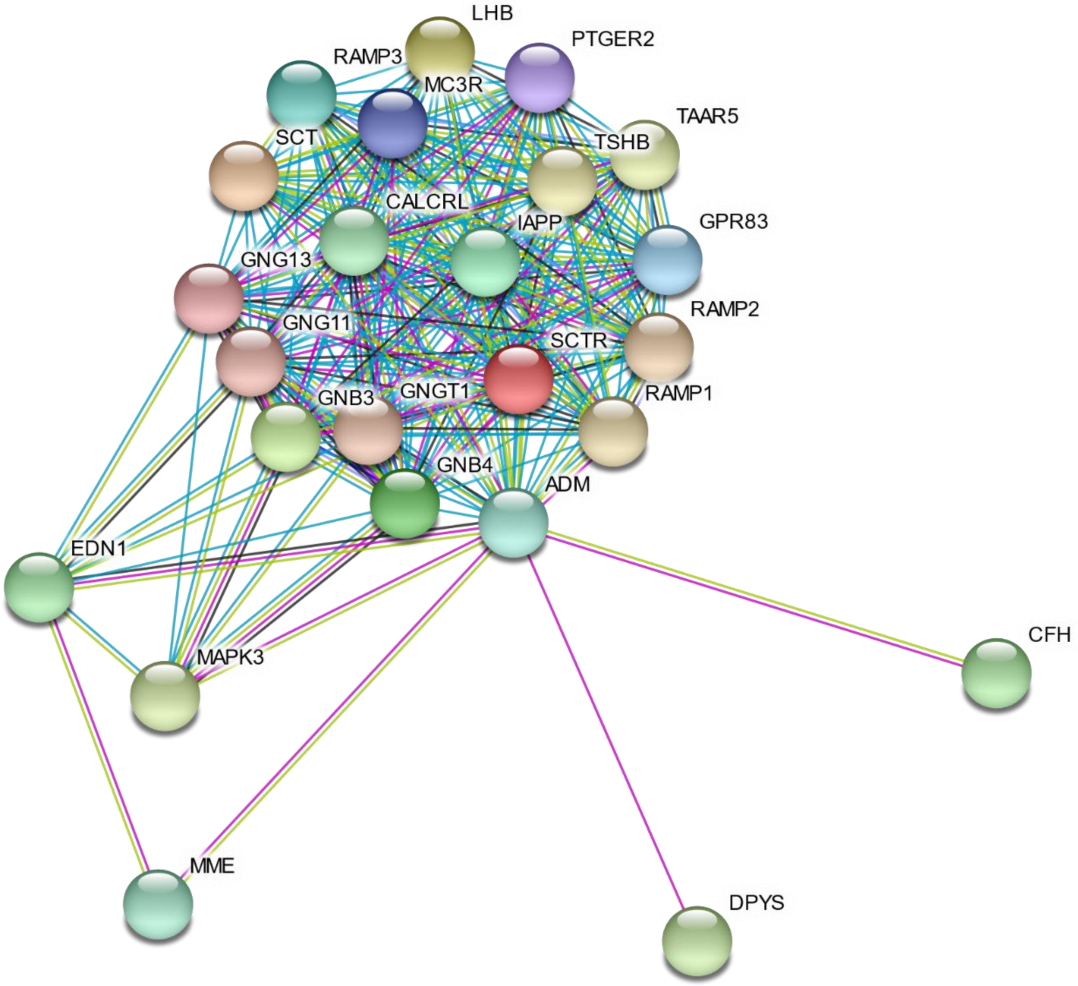

Supplementary Figure 2. Protein-protein interaction network of the top-scoring ADM-interacting proteins.

Supplementary Tables

Supplementary Table 1. Datasets used in the manuscript.

**Dataset ID**	**Platform**	**Sample n. and type**	**Normalization**	**Analysis**
BLUEPRINT	RNA-seq	119 hematopoietic cells	TPM	Hematopoietic cells and lineages
GSE98791	Agilent-021441	45 hematopoietic cells	Processed Signal intensities	Hematopoietic cells and lineages
GSE24759	Affymetrix HT-HG-U133A Early Access	158 hematopoietic cells	RMA	Hematopoietic cells and lineages
GSE24006	Affymetrix U133 Plus 2.0	23 AML and 23 hematopoietic cells at different stage of differentiation	RMA	AML *vs.* normal subpopulations
GSE63270	Affymetrix U133 Plus 2.0	41 AML and 35 hematopoietic cells at different stage of differentiation	MAS5	AML *vs.* normal subpopulations
GSE158596	RNA-seq	60 AML (blasts >85%) and 16 healthy G-CSF mobilized $CD34^+$ cells	Median of ratios	AML blasts *vs.* normal HSPC
GSE117090	Affymetrix HTA 2.0	58 AML and 14 hematopoietic cells at different stage of differentiation;<br>30 $CD8^+$ T from AML and 7 $CD8^+$ T from healthy	RMA	AML *vs.* normal subpopulations;<br>$CD8^+$ T from AML *vs.* healthy
GSE14924	Affymetrix U133 Plus 2.0	10 $CD4^+$ T from AML and 10 $CD4^+$ T from healthy	MAS 5.0	$CD4^+$ T from AML *vs.* healthy
NGS-PTL	Affymetrix HTA 2.0	61 AML BM MNCS (blasts ≥80%)<br>29 Ph− B-ALL MNCs	sst-RMA	AML *vs.* Ph− B-ALL;<br>AML and disease features; survival analyses
GSE14468	Affymetrix U133 Plus 2.0	459 AML BM/PB MNCs	RMA	AML and disease features
GSE6891	Affymetrix U133 Plus 2.0	68 AML BM MNCs; 410 AML PB MNCs	RMA	AML and disease features; survival analyses
GSE13159	Affymetrix U133 Plus 2.0	505 AML BM MNCs; 173 T-ALL MNCs;<br>441 B-ALL MNCs; 134 ALL NOS MNCs	RMA	AML *vs.* ALL;<br>AML and disease features
Beat AML	RNA-seq	142 AML BM MNCs; 87 AML PB MNCs;<br>19 normal BM MNCs	CPM (TMM)	AML and disease features; survival analyses
TCGA-LAML	RNA-seq	135 BM MNCs	CPM (TMM)	AML and disease features; survival analyses

BM: bone marrow; CPM: Counts Per Million; G-CSF: granulocyte colony-stimulating factor; MNCs: mononuclear cells; NOS: not otherwise specified; PB: peripheral blood; RMA: robust multi-array average; sst-RMA: signal space transformation RMA; TMM: Trimmed Mean of M values; TPM: Transcripts Per Million; *vs.*: *versus.*

Supplementary Table 2. Association between *ADM* expression levels and clinical/molecular data in the GSE13159 and NGS-PTL cohorts.

**Variable**	**GSE13159 (n=458)**			**NGS-PTL (n=61)**		
	n (%)	median [min-max]	*p*	n (%)	median [min-max]	*p*
**Age**	*NA*	*NA*	-			0.130
<60-years				17 (30.4)	20.9 [15.9-29.3]	
≥60-years				39 (69.6)	23.4 [15.2-67.5]	
missing				*5*		
**FAB**	*NA*	*NA*	-			0.468
M0				4 (11.4)	22.1 [18.9-29.3]	
M1				8 (22.9)	18.7 [15.2-39.1]	
M2				7 (20.0)	25.4 [16.1-58.4]	
M4				10 (28.6)	23.8 [19.2-55.5]	
M5				6 (17.1)	24.9 [16.4-56.3]	
M6				-		
M7				-		
missing				26		
**Cytogenetic group**			**<0.001**			0.076
t(8;21)	35 (7.6)	79.9 [13.6-584.1]		3 (4.9)	20.03 [19.73-24.65]	
inv(16)/t(16;16)	27 (5.9)	243.9 [25.8-1552.1]		2 (3.3)	32.05 [23.43-36.36]	
NK	-			31 (50.8)	20.55 [15.17-40.35]	
CK	45 (9.8)	393.4 [31.1-2033.9]		9 (14.8)	24.53 [16.05-36.28]	
KMT2A-r	29 (6.3)	51.3 [12.9-3541.1]		1 (1.6)	16.37	
Other	-			15 (24.6)	23.38 [18.71-30.18]	
Normal/Other*	322 (70.3)	186.8 [12.0-5712.9]		-		

* "NK" and "other" cytogenetic subgroups were not distinguished in GSE13159 dataset (CK: complex karyotype; *KMT2A*-r: *KMT2A*-rearranged; min-max: minimum-to-maximum value; mut: mutated; n: number; *NA*: not available; NK: normal karyotype; *p*: *p* value; *p*≤0.05 are highlighted as bold text).

frontiers

Supplementary Table 3. List of genes co-expressed with *ADM* in AML.

Gene	Pearson correlation coefficient	$\log_{10} p$
ADAM9	0.579	4.015
ANXA5	0.509	6.054
AQP9	0.640	8.551
ASAH1	0.574	5.736
BATF2	0.578	6.109
BCL2A1	0.576	4.710
BIRC3	0.638	9.134
BTG1	0.549	6.123
C1QA	0.749	5.471
C3	0.567	7.755
C5AR1	0.647	5.571
CARD16	0.672	7.557
CASP5	0.535	6.141
CCL2	0.568	10.073
CCL20	0.548	7.172
CD55	0.556	8.286
CD83	0.573	8.287
CDKN1A	0.558	9.767
CDKN2D	0.522	3.913
CEBPB	0.552	4.684
CHST7	0.650	6.838
CNEP1R1	0.526	5.071
CTSB	0.553	6.695
CTSH	0.557	5.156
CTSL	0.690	7.433
CTSS	0.603	6.756
CXCL10	0.572	10.280
CYBB	0.591	8.747
DIRC2	0.552	9.093
EDN1	0.663	5.238
EXT1	0.658	6.317
FAM20A	0.746	6.650
FCER1G	0.637	4.473
FCGR3A	0.689	13.723
FFAR2	0.644	12.890
FMNL2	0.694	5.708
FPR1	0.567	7.561
FTH1	0.636	7.889
FTH1P5	0.736	7.885
FTL	0.606	7.220
G0S2	0.590	10.802
GADD45B	0.599	9.033
GBP2	0.562	6.289
GCH1	0.576	7.226
GLTP	0.573	6.039
GNGT2	0.657	4.305
GNS	0.546	5.668
GPR137B	0.736	6.910
HBEGF	0.524	5.042
HCK	0.564	4.162
ID2	0.542	7.576
IER5	0.580	13.012
IFI30	0.628	13.143
IL10RA	0.640	8.606
IL21R	0.591	4.295
IL6	0.808	9.583
JUNB	0.583	8.895
LILRA1	0.573	5.487
LILRA5	0.742	8.385
LILRB1	0.742	8.416
LILRB2	0.689	9.583
LTA4H	0.552	4.627
LY96	0.654	4.996
LYN	0.597	7.968
MAFB	0.733	11.842
MARCO	0.787	5.500
MASTL	0.529	4.278
MBD2	0.713	8.557
METRNL	0.648	9.871
MYD88	0.569	6.706
MYOF	0.574	7.344
NAMPT	0.596	9.139
NFKBIZ	0.532	7.946
NINJ1	0.531	12.265
OTUD1	0.673	4.683
PELI1	0.638	7.992
PFKFB3	0.628	12.509
PHLDA2	0.562	9.342
PILRA	0.548	5.928
PLAUR	0.599	4.317
PLEKHO2	0.643	4.925
PLK2	0.525	4.312
PPT1	0.556	4.338
PRKACA	0.670	7.720
PSAP	0.670	6.291
PTP4A2	0.606	6.832
PTPRJ	0.573	7.196
RAB8B	0.554	5.483
RABGEF1	0.518	5.577
RAP2B	0.539	8.978
RASGEF1B	0.584	7.957
RASL11A	0.556	5.228
RASSF5	0.597	5.907
RGL1	0.599	4.405
RND3	0.726	6.862
RNF141	0.678	6.002
RNF144B	0.680	9.486
RRAS	0.637	6.713
SAT1	0.566	9.382
SDCBP	0.554	6.376
SECTM1	0.694	7.305
SERPINA1	0.668	7.137
SGK1	0.511	6.331
SGTB	0.601	4.775
SIDT2	0.675	5.748
SKIL	0.593	8.513
SLA	0.586	9.881
SLC15A3	0.626	9.988
SLC16A6	0.566	4.296
SLC2A3	0.539	8.925
SLC2A6	0.524	7.446
SLC43A2	0.578	5.484
SLC8A1	0.550	6.708
SLC9A9	0.656	5.088
SMPDL3A	0.684	7.176
SNORD89	0.550	6.345
SNX18	0.605	11.322
SOCS3	0.622	5.683
STX11	0.551	8.125
TAGLN	0.669	6.382
TBC1D8	0.547	6.610
TCF7L2	0.516	6.157
THEMIS2	0.504	5.691
TMEM127	0.582	7.441
TNFAIP3	0.641	10.042
TNFAIP6	0.619	5.150
TNFRSF1B	0.622	7.065
TNNT1	0.549	4.738
TPD52L2	0.543	3.952
TSPAN14	0.617	7.650
UPP1	0.534	9.548
VASP	0.578	5.388
ZFAND5	0.542	6.582
ZFP36L1	0.612	9.440
ZNF703	0.652	4.244

Pearson correlation coefficient and p represent the weighted arithmetic mean of the correlation coefficient and of the p value across the datasets, respectively.

Supplementary Table 4. Pathway enrichment analysis of genes co-expressed with *ADM* in AML.

	Colle-ction	Term	Adj-*p*	Genes
**Immune response and inflammation**	React	Immune System	3.12E-06	C1QA;CDKN1A;C5AR1;LY96;TNFAIP3;PTPRJ;LILRA1;IFI30;CTSS;LILRA5;C3;SOCS3;FCGR3A;CTSL;CTSH;PRKACA;GBP2;CD55;CTSB;VASP;LYN;FCER1G;CYBB;LILRB1;LILRB2;TNFRSF1B;HCK;RNF144B;IL6;PELI1;MYD88;HBEGF;BIRC3
	GO-BP	regulation of immune response	3.68E-02	C3;LYN;FCGR3A;LILRB1;PILRA;LILRA1;LILRB2;BIRC3
	GO-BP	leukocyte differentiation	1.31E-02	LYN;RRAS;LILRB1
	React	Adaptive Immune System	1.52E-04	VASP;LYN;CDKN1A;CYBB;PTPRJ;LILRB1;LILRA1;LILRB2;IFI30;CTSS;LILRA5;C3;SOCS3;RNF144B;FCGR3A;CTSL;CTSH;PRKACA;CTSB;HBEGF
	React	Innate Immune System	1.06E-03	LYN;C1QA;CDKN1A;FCER1G;C5AR1;LY96;TNFAIP3;CTSS;C3;HCK;FCGR3A;CTSL;PELI1;PRKACA;MYD88;CD55;CTSB;HBEGF;BIRC3
	KEGG	Antigen processing and presentation	2.36E-02	CTSL;IFI30;CTSS;CTSB
	GO-BP	dendritic cell differentiation	4.15E-02	LYN;BATF2;LILRB1
	GO-BP	regulation of defense response	2.06E-02	HCK;CASP5;TNFAIP3;MYD88;BIRC3
	GO-BP	cellular response to molecule of bacterial origin	3.40E-06	CXCL10;IL6;TNFAIP3;ADAM9;CCL2;LILRB1;CARD16;LILRB2;TNFRSF1B
	GO-BP	pattern recognition receptor signaling pathway	3.23E-04	CTSL;LY96;FFAR2;CTSS;MYD88;CTSB
	GO-BP	immune response-regulating cell surface receptor signaling pathway	8.39E-03	LYN;LILRB1;LILRB2
	KEGG	Toll-like receptor signaling pathway	4.64E-02	CXCL10;IL6;LY96;MYD88
	GO-BP	toll-like receptor signaling pathway	5.21E-03	CTSL;LY96;CTSS;MYD88;CTSB;BIRC3
	React	Toll-Like Receptors Cascades	1.78E-02	CTSL;PELI1;LY96;MYD88;CTSS;CTSB;BIRC3
	React	Trafficking and processing of endosomal TLR	2.09E-02	CTSL;CTSS;CTSB
	GO-BP	neutrophil activation involved in immune response	3.20E-13	ASAH1;SERPINA1;TNFAIP6;C5AR1;FPR1;PTPRJ;SLC2A3;GNS;CTSS;C3;SDCBP;FTH1;PSAP;CTSH;LTA4H;CD55;CTSB;FCER1G;PLAUR;CYBB;LILRB2;TNFRSF1B;TSPAN14;RAP2B;PLEKHO2;FTL
	GO-BP	neutrophil mediated immunity	1.12E-13	ASAH1;SERPINA1;TNFAIP6;C5AR1;FPR1;PTPRJ;SLC2A3;GNS;CTSS;C3;SDCBP;FTH1;PSAP;CTSH;LTA4H;CD55;CTSB;FCER1G;PLAUR;CYBB;LILRB2;TNFRSF1B;IL6;TSPAN14;RAP2B;PLEKHO2;FTL

	GO-BP	neutrophil degranulation	3.93E-13	ASAH1;SERPINA1;TNFAIP6;C5AR1;FPR1;PTPRJ;SLC2A3;GNS;CTSS;C3;SDCBP;FTH1;PSAP;CTSH;LTA4H;CD55;CTSB;FCER1G;PLAUR;CYBB;LILRB2;TNFRSF1B;TSPAN14;RAP2B;PLEKHO2;FTL
	KEGG	Phagosome	1.06E-02	C3;MARCO;FCGR3A;CTSL;CYBB;CTSS
	KEGG	Fc gamma R-mediated phagocytosis	3.85E-02	LYN;VASP;HCK;FCGR3A
	KEGG	Complement and coagulation cascades	9.67E-04	C3;C1QA;SERPINA1;C5AR1;PLAUR;CD55
	GO-BP	inflammatory response	3.93E-04	LYN;CXCL10;IL6;CEBPB;TNFAIP6;CCL20;C5AR1;FPR1;CYBB;CCL2;TNFRSF1B
	GO-BP	regulation of acute inflammatory response	2.20E-02	C3;C1QA;IL6;C5AR1;FFAR2;CD55
	GO-BP	regulation of inflammatory response	3.83E-03	HCK;TNFAIP6;CASP5;TNFAIP3;FFAR2;METRNL;MYD88;BIRC3
	GO-BP	regulation of interleukin-6 production	2.21E-02	IL6;TNFAIP3;LILRB2;MYD88
**Cellular response to stimuli**	KEGG	Chemokine signaling pathway	6.39E-03	LYN;CXCL10;HCK;GNGT2;CCL20;CCL2;PRKACA
	GO-BP	cellular response to cytokine stimulus	3.23E-03	CDKN1A;CCL20;IL10RA;FPR1;TNFRSF1B;ZFP36L1;SOCS3;CXCL10;HCK;IL6;CCL2;PRKACA;JUNB
	KEGG	Cytokine-cytokine receptor interaction	4.10E-02	CXCL10;IL6;CCL20;IL10RA;IL21R;CCL2;TNFRSF1B
	GO-BP	lipopolysaccharide-mediated signaling pathway	2.07E-02	LYN;HCK;CCL2
	GO-BP	response to lipopolysaccharide	5.30E-07	CXCL10;IL6;GCH1;ADAM9;LY96;TNFAIP3;CCL2;LILRB1;CARD16;LILRB2;TNFRSF1B;JUNB
	GO-BP	cellular response to lipopolysaccharide	3.34E-08	LYN;HCK;CXCL10;IL6;TNFAIP3;ADAM9;CCL2;LILRB1;CARD16;LILRB2;TNFRSF1B
	GO-BP	cytokine-mediated signaling pathway	1.37E-04	CDKN1A;CCL20;IL10RA;FPR1;IFI30;TNFRSF1B;SOCS3;CXCL10;HCK;IL6;PELI1;IL21R;CCL2;PRKACA;GBP2;JUNB;MYD88;BIRC3
	GO-BP	cellular response to lipid	2.22E-06	LYN;CXCL10;IL6;ZNF703;ADAM9;TNFAIP3;CCL2;FFAR2;LILRB1;CARD16;LILRB2;TNFRSF1B
	GO-BP	response to lipid	8.48E-03	CDKN2D;CXCL10;GCH1;LY96;CTSH;TNFRSF1B;JUNB
**Signaling pathways**	KEGG	NF-kappa B signaling pathway	2.83E-04	LYN;BCL2A1;GADD45B;TNFAIP3;LY96;MYD88;BIRC3
	KEGG	NOD-like receptor signaling pathway	5.40E-06	IL6;CASP5;NAMPT;CYBB;TNFAIP3;CCL2;CARD16;GBP2;MYD88;CTSB;BIRC3
	KEGG	FoxO signaling pathway	6.08E-03	CDKN2D;IL6;CDKN1A;GADD45B;PLK2;SGK1
	KEGG	HIF-1 signaling pathway	1.00E-02	IL6;CDKN1A;EDN1;PFKFB3;CYBB
	KEGG	JAK-STAT signaling pathway	4.56E-02	SOCS3;IL6;CDKN1A;IL10RA;IL21R
	KEGG	IL-17 signaling pathway	1.36E-03	CXCL10;CEBPB;IL6;CCL20;TNFAIP3;CCL2
	KEGG	TNF signaling pathway	6.53E-08	SOCS3;CXCL10;IL6;EDN1;CEBPB;CCL20;TNFAIP3;CCL2;TNFRSF1B;JUNB;BIRC3

**Cell growth**	GO-BP	regulation of cell growth	4.74E-02	CDKN2D;SDCBP;CDKN1A;BTG1;PPT1;PTPRJ;SGK1
	GO-BP	regulation of cell proliferation	1.56E-07	LYN;CDKN2D;TCF7L2;CDKN1A;EDN1;BTG1;TBC1D8;SLA;PTPRJ;TNFRSF1B;ZFP36L1;SDCBP;CXCL10;HCK;IL6;TMEM127;FTH1;NAMPT;ZNF703;CTSH;SGK1;IER5;JUNB;HBEGF
	GO-BP	negative regulation of lymphocyte proliferation	7.82E-03	LYN;CEBPB;LILRB1;LILRB2
**Cellular senescence and cell death**	GO-BP	regulation of nitric-oxide synthase biosynthetic process	1.04E-02	EDN1;NAMPT;CCL2
	GO-BP	cellular response to oxygen-containing compound	2.53E-05	LYN;TNFAIP3;LILRB1;LILRB2;TNFRSF1B;ZFP36L1;CXCL10;IL6;ZNF703;ADAM9;CCL2;FFAR2;CARD16
	KEGG	Ferroptosis	3.86E-03	FTH1;CYBB;SAT1;FTL
	KEGG	Apoptosis	1.64E-03	GADD45B;BCL2A1;CTSL;CTSH;CTSS;CTSB;BIRC3
	GO-BP	regulation of apoptotic process	1.71E-02	GADD45B;BCL2A1;PLK2;ANXA5;PLAUR;LILRB1;TNFRSF1B;HCK;RNF144B;IL6;PPT1;CTSH;SGK1;MYD88;CTSB;BIRC3
	GO-BP	negative regulation of apoptotic process	3.71E-04	CDKN2D;BCL2A1;PLK2;ANXA5;PLAUR;TNFRSF1B;HCK;RNF144B;IL6;PPT1;CTSH;CCL2;CARD16;MYD88;BIRC3
	GO-BP	negative regulation of programmed cell death	1.51E-02	HCK;RNF144B;IL6;BCL2A1;PLK2;ANXA5;PPT1;PLAUR;CTSH;MYD88;BIRC3
	GO-BP	negative regulation of cysteine-type endopeptidase activity involved in apoptotic process	1.45E-02	CDKN2D;PLAUR;CARD16;TNFRSF1B;BIRC3
	KEGG	Cellular senescence	1.18E-02	IL6;CDKN1A;RRAS;GADD45B;RASSF5;ZFP36L1
	KEGG	Necroptosis	4.42E-02	FTH1;CYBB;TNFAIP3;FTL;BIRC3

Adj: adjusted; GO-BP: Gene Ontology Biological Processes; React: Reactome.
